# Self-Rated Benefits of Auditory Performance after Bonebridge Implantation in Patients with Conductive or Mixed Hearing Loss, or Single-Sided Deafness

**DOI:** 10.3390/life12020137

**Published:** 2022-01-18

**Authors:** Anna Ratuszniak, Piotr H. Skarzynski, Elżbieta Gos, Henryk Skarzynski

**Affiliations:** 1 Otorhinolaryngology Surgery Clinic, World Hearing Center, Institute of Physiology and Pathology of Hearing, 02-042 Warsaw, Poland; h.skarzynski@ifps.org.pl; 2 World Hearing Center, Department of Teleaudiology and Screening, Institute of Physiology and Pathology of Hearing, 02-042 Warsaw, Poland; p.skarzynski@csim.pl (P.H.S.); e.gos@ifps.org.pl (E.G.); 3 Heart Failure and Cardiac Rehabilitation Department, Faculty of Medicine, Medical University of Warsaw, 02-091 Warsaw, Poland; 4 Institute of Sensory Organs, 05-830 Kajetany, Poland

**Keywords:** bone conduction implant, Bonebridge, APHAB, self-related benefits, hearing implant

## Abstract

The Bonebridge implant can be a satisfactory solution for patients with conductive or mixed hearing loss (CHL or MHL), or with single-sided deafness (SSD). The aim of the study was to assess patients’ self-reported benefits with the Bonebridge and characterize the relationships between pre-implantation audiometric data, auditory functioning, and satisfaction after implantation. A focus was to see whether different types of hearing loss were associated with particular benefits. The study sample consisted of 81 patients. Procedures comprised pure tone audiometry before implantation, the Abbreviated Profile of Hearing Aid Benefit (APHAB) questionnaire, and a structured interview asking about satisfaction. Statistically significant improvements after implantation were found in all groups (CHL, MHL, SSD) on the APHAB questionnaire. In the structured interview, patients with SSD were the least satisfied. No significant correlation was found between pre-operative air-bone gap and bone conduction thresholds or with APHAB score. Bonebridge implantation is beneficial to patients with CHL or MHL, or with SSD. Assessment of patients for Bonebridge implantation is complex, and audiometric data should be complemented by patient-reported outcomes to provide deeper insight into their individual needs and attitudes.

## 1. Introduction

The World Health Organization estimates that hearing loss currently affects more than 1.5 billion people or 20% of the global population [1]. For people affected by this problem, hearing loss means limitations in activity and restrictions to social participation, mainly caused by difficulties in verbal communication, and can have negative effects on educational, social, and professional aspects of their lives. Hearing loss can, depending on many factors, negatively impact quality of life; in most cases, undertaking effective interventions makes it possible to reduce the negative impacts. Two forms of intervention are hearing aids and implantable devices. The effectiveness of an implant in improving auditory functioning can be assessed by means of audiological tests carried out in a clinic. The most frequently used methods include assessment of pure-tone thresholds and speech discrimination in quiet and noise under unaided and aided conditions. However, the idea of a patient-centered approach has promoted interest in the patient’s subjective assessment of hearing loss outcomes to complement the objective measurements.

The subjective auditory benefit gained from an intervention can be evaluated by researchers or clinicians using a range of different patient-reported outcome measures (PROMs). These include the APHAB, the Glasgow Benefit Inventory (GBI), the International Outcome Inventory for Hearing Aids (IOI-HA), the Speech, Spatial, and Qualities of Hearing (SSQ), the Hearing Device Satisfaction Scale (HDDS), and the Bern Benefit in Single-Sided Deafness (BBSS).

On the basis of his own and related research, Cox has distinguished seven categories of self-reported outcome data: benefit, satisfaction, quality of life, use, impact on others, residual participation restrictions, and residual activity limitations [2]. A measure of benefit quantifies the change in a hearing-related dimension of functioning provided by the hearing prosthesis. However, self-reported outcome measures are multidimensional and so different domains do not necessarily correlate with each other. Cox also underlines that it is important to pay careful attention to which domains are being measured by a particular inventory and that it is essential to define the goals of the measurement and the intended uses of the data.

Subjective benefit is typically measured in one or two dimensions: activity limitations and participation restrictions. One example of a measuring tool for residual activity limitation is the APHAB questionnaire [3].

Magele et al. [4], in their meta-analysis of active transcutaneous bone conduction implants, showed that APHAB stands out as the most frequently used tool. This questionnaire is used to quantify the disability associated with a hearing loss and the reduction of disability achieved with a hearing prosthesis [3]. APHAB makes it possible to assess the limitations in activity and the restrictions in participation caused by hearing loss (both with and without the prosthesis) and then calculate the benefit.

In addition to gauging the auditory benefit of using a selected questionnaire, it is also important to assess the patient’s satisfaction with the medical intervention. Satisfaction is the aggregate of the individual physical, social, and psychological changes resulting from the use of a hearing prosthesis [2]. Based on data in the literature, there are several important elements affecting satisfaction [5]. Cox categorized them into six domains: cosmetic and self-image, sound quality, benefit, comfort and ease of use, cost, and service. Presumably, the importance of each domain depends on the individual.

In the present work, we assess the impact that one medical intervention–the Bonebridge (BB) system–had on the hearing functioning of three groups of patients: those with conductive hearing loss (CHL), mixed hearing loss (MHL), and single-sided deafness (SSD).

The BB active bone conduction implant (Med-El, Innsbruck, Austria) is used in patients with conductive or mixed hearing loss, of various etiology, who have bone conduction thresholds not exceeding 45 dB HL, in the frequency range 500–3000 Hz. BB can also be a solution for patients after radical modified surgery or after unsuccessful tympanoplasty (with the aid of bioactive glass) [6,7,8]. Moreover, the BB implant can also be used in single-sided deafness in an ear with severe to profound hearing loss where the contralateral ear has normal hearing [9,10,11]. Another recent solution used in this type of hearing loss is the cochlear implant [12]. The decision on what solution is used depends on audiological and anatomical factors, the etiology of hearing loss, and other factors. The BB system was introduced into clinical practice in 2012. Since then, significant benefits have been reported in the literature about how it can improve hearing and speech discrimination, both in children and adults [10,12,13,14,15,16,17], and improve daily functioning, as reported by patients through appropriate questionnaires (PROMs) [4,10,13,14,15,16,17,18,19,20,21,22].

The aim of the study was to assess the self-reported benefits after implantation of the BB system and explore the relationships between pre-implantation audiometric data and perceived benefits and satisfaction. Efforts were also made to determine whether patients with different types of hearing loss (CHL, MHL, SSD) experienced different benefits and levels of satisfaction.

## 2. Materials and Methods

### 2.1. Subjects

The APHAB questionnaire and structured interview were sent via post to 151 BB users who underwent surgical intervention with the BB system between 2012 and 2019. The interview and questionnaire were returned by 103 patients, a response rate of 68%.

There were 12 children who were excluded, as well as 10 other subjects who used the audio processor for less than 4 h per day. The final study sample comprised 81 adult subjects.

There were 39 women and 42 men, aged from 18 to 74 years. Patients were stratified according to their type of hearing loss. There were 25 patients who had CHL, 36 patients who had MHL, and 20 who had SSD. Statistically significant differences in terms of age, use of a processor (hours/day), and length of use (years) were found. The MHL patients were significantly older than the CHL patients; they used a processor more hours per day than the SSD patients and had used their BB for more years than all other patients.

In the CHL group, 13 patients (52%) had unilateral, and 12 (48%) had bilateral, hearing loss. In the MHL group, the figures were 12 (33.3%) and 24 (66.7%), respectively. The difference was not statistically significant (*χ*^2^ = 2.13; *p* = 0.145).

PTA4 for air conduction in the implanted ear was *M* = 61.35 dB HL (*SD* = 10.38) in the CHL group; *M* = 75.17 dB HL (*SD* = 13.70) in the MHL group; and *M* = 111.94 dB HL (*SD* = 12.93) in the SSD group. In the SSD group, the opposite ear was a normal hearing ear with air conduction thresholds ≤ 20 dB HL.

PTA4 for bone conduction in the implanted ear was M = 14.42 dB HL (SD = 3.47) in the CHL group, and M = 33.51 dB HL (SD = 9.95) in the MHL group.

The socio-demographic characteristics of the patients are shown in Table 1.

In determining relationships between APHAB results and audiometric data (such as bone-conduction thresholds and air-bone gap), the CHL and MHL groups were combined.

Before making the decision to implant, patients underwent a brief trial with a bone conduction device on a softband. The trial allowed an assessment to be made of the potential benefits from implantation and also gave the patient the opportunity to experience the sort of sensations an implant would provide.

### 2.2. Surgery

In all cases, surgery was conducted under general anesthesia following standard procedures suggested by the manufacturer of the device. The BCI 601 implant was placed in the sinodural angle in all cases. In the first step, a standard S-shape cut was made, as with a typical cochlear implant. The subcutaneous tissue was opened with a monopolar coagulator and a cut made in the periosteum parallel to the skin cut. The periosteum was carefully dissected to preserve tissue. Then, a cavity was made for the device with a cutting or diamond burr, and the dimensions of the cavity checked against the template provided with the implant. The implant was put in place, and small holes for the dedicated screws were made with a single-use burr. There was no need for a lift, which can sometimes be placed between the wing of the implant and the bone if space is lacking. After placing the implant in the correct position, three layers of sutures were added, and a strong dressing was applied to avoid hematoma. All surgeries were uneventful.

### 2.3. Pre-Operative Audiometric Assessment

**Figure 1:**
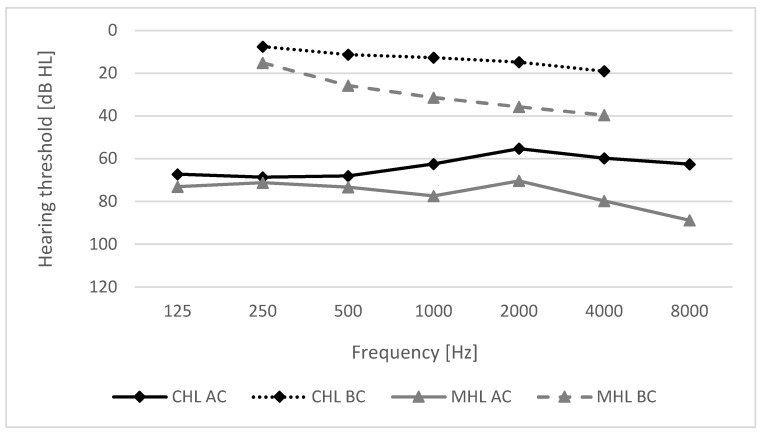
Mean hearing thresholds (air conduction, AC; bone conduction, BC) in the implanted ear in patients with conductive (CHL) and mixed hearing loss (MHL).

Pure-tone audiometry was performed in an anechoic chamber with an Otometrics Madsen Itera II diagnostic audiometer. The air conduction threshold was measured with on-ear headphones TDH39 for 125–8000 Hz; bone conduction was measured using a calibrated bone transducer B-71 for 250–4000 Hz. Measurements were performed the day before implantation of the BB system. The audiological details for the CHL and MHL groups are presented in Figure 1.

### 2.4. Post-Operative Assessment

The Abbreviated Profile of Hearing Aid Benefit (APHAB) [3] was given to the patients following at least 3 months of use of the BB system. APHAB is the most widely used hearing-specific questionnaire [23]. It measures (in percentages) activity limitations and participation restrictions due to hearing loss under unaided and aided conditions. APHAB consists of 24 questions which are divided into four sub-scales: ease of communication (EC), background noise (BN), reverberation (RV), and aversiveness (AV). A Global Score (GS) was calculated as the mean of the first three categories. Cox and Alexander suggested that a difference between unaided and aided scores of at least 22 points on the EC, BN, and RV subscales was needed to be reasonably certain that the scores represented a real difference between conditions [3].

To investigate patient satisfaction with the BB system, a structured interview was conducted. Three questions were asked to probe general satisfaction based on two of the six elements described by the Cox–sound quality and benefit. Responses were collected on a 5-point Likert scale (Table 2).

### 2.5. Statistical Analysis

The sociodemographics of the study groups were examined using descriptive statistics and percentages. A chi-square test or one-way analysis of variance (ANOVA) was used to evaluate differences between the CHL, MHL, and SSD groups. A mixed-design analysis of variance was applied to compare difficulties in hearing (APHAB outcomes) among the CHL, MHL, and SSD groups under aided and unaided conditions (with BB on and off). Relationships between difficulty in hearing and air-bone gap, bone conduction, duration of BB use (years), and duration of the processor use (hours/day) were established using a rho-Spearman correlation coefficient. A two-sided *p*-value < 0.05 was considered to indicate statistical significance. SPSS IBM Statistics software (v. 24) was used for analysis.

### 2.6. Study Design

This study was an observational cross-sectional design with a questionnaire and structured interview form sent by post. The APHAB questionnaire and structured interview were given to each implanted patient to obtain information about auditory functioning and satisfaction with the BB system. All subjects signed an informed consent. The study protocol was approved by the Institutional Review Board of the Institute of Physiology and Pathology of Hearing (KB.IFPS:21/2018) and conformed with the Declaration of Helsinki.

## 3. Results

### 3.1. Subjective Assessment of Satisfaction—Results of the Structured Interview

**Figure 2:**
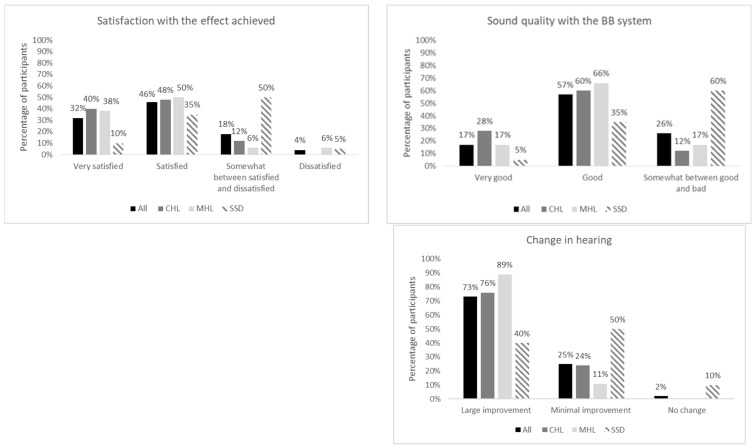
Structured interview outcomes—frequency of occurrence (%).

Most of the patients reported very good or good hearing (sound quality) with the BB implant. They were generally satisfied with the achieved effect and noticed significant improvement. Subjective evaluation showed statistically significant differences between the three groups. Generally, the SSD patients rated their present hearing as not as good as that of the CHL and MHL patients, and they were less satisfied with the BB’s performance. Furthermore, the SSD patients assessed their hearing improvement (benefit) as being smaller than that of the CHL or MHL patients. All differences between the three groups were statistically significant (*p* < 0.01). Detailed structured interview outcomes are given in Figure 2.

### 3.2. Limitation in Activity and Restriction in Participation—APHAB Outcomes

**Figure 3:**
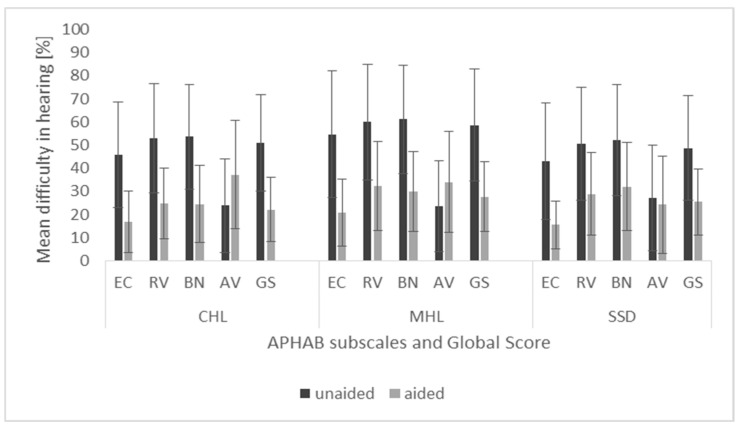
Unaided and aided scores of the APHAB questionnaire. EC, Ease of Communication; RV, Reverberation; BN, Background Noise; AV, Aversiveness; GS, global score. All differences in the subscales and global scores are statistically significant. The bars are mean scores, the error bars are standard deviations.

Mean scores and standard deviations for the APHAB subscales and global scores obtained under unaided and aided conditions in the three groups of patients are shown in Figure 3. Statistically significant changes were revealed in the APHAB outcomes; however, only the effect of aided vs. unaided was statistically significant: for the EC subscale *F* = 123.61, *p* < 0.001, *e*^2^ = 0.61; for the RV subscale, *F* = 96.49, *p* < 0.001, *e*^2^ = 0.55; for the BN subscale, *F* = 107.22, *p* < 0.001, *e*^2^ = 0.58; for the AV subscale, *F* = 6.61, *p* = 0.012, *e*^2^ = 0.08; and for the APHAB global score, *F* = 139.80, *p* < 0.001, *e*^2^ = 0.64. In general, the effect was that hearing difficulty significantly decreased with BB use in all three groups. An interaction effect was statistically non-significant, so the above-mentioned changes between unaided and aided conditions were observed no matter which group (CHL, MHL, or SSD) the patients belonged to. A group effect was also statistically non-significant.

The changes in APHAB scores are shown in Figure 3.

An improvement of at least 22 points in the EC subscale was found in 57% of patients (in 52% of patients in the CHL group, 67% in the MHL group, and 55% in the SSD group). A difference of at least 22 points in the RV subscale was found in 46% of patients (in 52% of the patients in the CHL group, 47% in the MHL group, and 35% in the SSD group). A difference of at least 22 points in the BN subscale was found in 41% of patients (in 56% of the patients in the CHL group, 58% in the MHL group, and 30% in the SSD group).

### 3.3. Relationship between APHAB and Hearing Impairment in the Contralateral Ear

A check was made whether hearing impairment in the contralateral ear had an impact on limitations in functioning and activity after BB implantation. Patients with CHL or MHL were divided into three subgroups depending on the hearing loss in their contralateral ear: those with normal hearing, with mild/moderate hearing loss, and with severe/very severe hearing loss. The division into degree of hearing loss was made according to the Bureau International d’Audiophonologie (BIAP) classification [24]. It was found that, before implantation, hearing difficulty was, on average, 40.54, 60.73, and 77.09 points, respectively, and decreased significantly after BB implantation. The difference between the GS for the unaided condition between normal hearing and severe hearing loss in the opposite ear was 37 points, while for the aided condition, it was only 12 points. For both conditions, when the degree of hearing loss in the opposite ear was greater, the GS value was also higher, as can be seen in Table 3.

### 3.4. Relationships between APHAB Score, Air-Bone Gap, and Bone Conduction Threshold in the Implanted Ear

**Figure 4:**
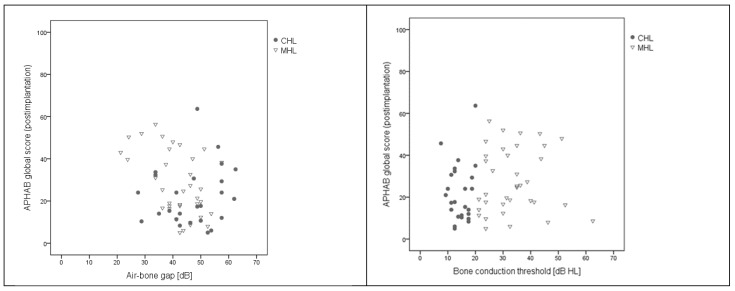
Scatter plot of global APHAB score and air-bone gap (**left**) and of global APHAB score and bone conduction threshold (**right**). In both plots, the correlations were not significant.

The correlation between APHAB global score and air-bone gap was statistically non-significant: *rho* = 0.21; *p* = 0.100 in the CHL and MHL group. Similarly, the correlation between APHAB global score and bone conduction was statistically non-significant (*rho* = −0.21; *p* = 0.112), as can be seen in Figure 4.

### 3.5. Relationships between APHAB Score, Duration of BB Use, and Time per Day of Processor Use

We did not find any significant correlation between difficulty in hearing measured with APHAB and duration of implant use. The relationship between APHAB global score and duration of BB use (in years) was *rho* = 0.09; *p* = 0.493 in the CHL and MHL group. The correlation was *rho* = 0.26; *p* = 0.268 in the SSD group.

The relationship between hours of processor use per day and APHAB global score was *rho* = 0.01; *p* = 0.969 in the CHL and MHL group. The correlation was *rho* = −0.04; *p* = 0.863 in the SSD group.

## 4. Discussion

### 4.1. Conductive and Mixed Hearing Loss

The aim of our study was to determine the benefits to patients who had different types of hearing loss and used the BB system. PROMs outcomes indicated significant benefit after BB implantation in patients with conductive and mixed hearing loss. Many previous studies have given similar results [10,14,15,17,18,25]. The APHAB results in our study can be directly compared with those of Yang et al., who reported on 100 patients with bilateral microtia–atresia, which is the largest group of BB users with conductive and mixed hearing loss in the literature [14]. In BB users with microtia, a reduction of problems with auditory functioning in everyday life (GS) was about 35 percentage points, while in our work, it was about 30 points, which is fairly comparable. Our population was considerably more mixed than that in the Yang et al. study, which could explain the slightly smaller benefit. Additionally, we have demonstrated that, although there was an overall benefit, on the AV subscale, patients aided with the BB scored higher than in the unaided condition, indicating that some discomfort might be present. A similar effect has been noted by other authors [19]. Most patients were satisfied or very satisfied with BB intervention (94% of cases), and moreover, they assessed their hearing under the aided condition as good or very good (above 60%). More than 76% of treated cases achieved an improvement of large or very large (above 76%). Every person in this group noticed a change after using the BB implant. The estimate of patient satisfaction achieved with the BB, which was probed by APHAB, was largely related to their limitations in activity and restrictions in participation. One needs to bear in mind that this relationship is not a simple one, and that satisfaction is clearly a complex variable, which includes elements that are not often explicitly addressed since most effort is devoted to documenting a reduction in disability.

No significant correlation was observed between the audiological parameters obtained in the audiometry tests (before implantation of the BB system) and the achieved benefits reported in the APHAB questionnaire (after implantation). However, the level of hearing in the opposite ear in CHL and MHL patients seems to be interesting and important. The greater the hearing loss in the opposite ear, the greater the problems observable in everyday functioning under unaided conditions. It can be assumed that a patient qualified for a BB according to the set criteria will achieve significant benefits in auditory functioning after implantation. However, the bone conduction hearing thresholds, the size of the air-bone gap, and the hearing condition in the opposite ear at the qualifying stage for surgery do not provide any guidance as to the degree of subjective benefits obtained after surgery (in terms of both limitations in functioning and activity, and in satisfaction).

This suggests that it is not the selected pre-operative audiological parameters, but some other internal and external factors that are key. For example, the individual’s needs, expectations, experience in using a hearing prosthesis, acoustic conditions, and the patient’s home environment can all have a significant impact on the change in functioning after BB implantation.

### 4.2. Single-Sided Deafness

The basic consequences of SSD hearing loss include problems with localizing a sound source, discriminating speech in noise, and comfortable hearing under various acoustic conditions, all of which affect the level of perceived disability [26,27]. For patients with SSD, localization of a sound source when using a bone conduction device is still limited, but there are benefits in terms of functioning in noise [27,28]. Using the APHAB questionnaire, Saroul et al. reported the benefits of a bone conduction device in a group of 36 SSD patients [29]. The average decrease in GS was comparable to that obtained in this study. Concerning satisfaction, the majority of our patients rated their hearing (sound quality) with the BB device as somewhere between good and bad (60%), half the group assessed their satisfaction as between satisfied and dissatisfied, 50% regarded the improvement obtained as minimal, and 40% as large. In two cases, no change in hearing after implantation was observed. The SSD patients had a lower overall level of satisfaction with the BB than did the CHL or MHL patients. Moreover, the SSD patients perceived the improvement in their hearing (benefit) as smaller than did the CHL or MHL patients. These findings correspond with the everyday experience and observations from the authors’ clinical practice: when directly asked about benefits and levels of satisfaction, SSD patients seem to be less satisfied than those with CHL or MHL.

It is worth noting that normal hearing in the opposite ear plays a key role in SSD patients implanted with a bone conduction system. These devices work by contralaterally routing the signal (CROS) to the normal hearing side. The qualification criteria provide that the air conduction hearing thresholds in the better ear should be no worse than 20 dB HL. If the hearing in this ear deteriorates, as it might for physiological reasons in the elderly, it is expected that the effectiveness of this type of device will become progressively lower. In making a long-term prognosis, this risk should be considered a limitation of bone conduction devices of the CROS type.

Recently, an increasingly common approach to patients with SSD is to use a cochlear implant [30,31]. Based on APHAB results, Skarzynski et al. have shown, in a comparable group of SSD patients, a GS benefit at the level of 16 percentage points [32]. Távora-Vieira et al. report approximately 24 percentage points [33]. By comparison, in our work, there was a gain of about 23 percentage points in GS. This means that in a group of SSD patients, it is possible to obtain comparable results, as subjectively assessed by an APHAB questionnaire, as in a group of cochlear implant users. The pre-operative situation of SSD patients qualified for a CI differs from those seeking a BB because, with a CI, there is no way of performing a pre-operative test to estimate the likely levels of post-operative perception and satisfaction. This might result in SSD patients becoming dissatisfied with their CI because of non-realistic expectations. In our clinical practice, we offer SSD patients all currently available solutions—cochlear implant, bone-anchored hearing aid, bone conduction implant, or a contralateral routing of the sound system. Although in SSD, the questionnaire results and the degree of satisfaction with the hearing prosthesis varied, it can be said that bone conduction devices appear to be effective in reducing the limitations in activity and restrictions in participation about which SSD patients complain, as other studies have confirmed [34].

### 4.3. Limitations

A limitation of collecting data through a postal questionnaire is not being sure of how representative the study group is. Non-return of a questionnaire can involve many factors, so it is important to try to contact the person by phone. Better chances of eliciting an answer come from ringing beforehand, attaching a stamped return envelope, providing written information about the survey, and including information about the funding of the survey, all of which affect its credibility. Following up with a reminder call is often effective. Even with the use of these tools, the response rate in our study was only 68%. A limitation of methods requiring indirect feedback from patients is its reliance on the patient’s cooperation. Wong et al. comment that “For healthcare research, those who are less satisfied (Nguyen et al., 1983) and have attained lower educational standing, income, and employment are less likely to respond (Draper and Hill, 1996). In contrast, Ware et al. (1983) reported more satisfied patients were less likely to return a satisfaction questionnaire. The discrepancies are probably related to the type of healthcare being evaluated, whether the patients believe that the responses provided would modify the care they receive and the outcome, and the reasons causing their dissatisfaction” [35]. Of course, not all the factors in this type of research are controllable, and many are random.

### 4.4. Summary

As far as the authors know, there is no study comparing outcomes of BB implantation in patients with CHL, MHL, and SSD in terms of self-assessment. If one assumes that each of these types of hearing loss involves a different type of auditory problem, our efforts were directed to investigating whether, after using the BB implant, each group would report different ratings on the auditory functioning scale, as well as on the satisfaction scale.

According to WHO, the current classification of hearing loss means that only adults with a permanent unaided hearing impairment of more than 40 dB HL in the better ear are regarded as having a disabling hearing impairment [36]. That definition of disabling hearing impairment excludes all people with SSD. However, our questionnaire study indicates that people with SSD have clear difficulties in auditory functioning. Indeed, the degree of self-reported problems in the unaided condition is similar to that obtained in people with CHL or MHL. At the same time, for aided conditions, there were no statistically significant differences in reported problems between the three groups of patients. Notwithstanding, in the structured interview, some statistically significant differences were found. The SSD patients rated their present hearing as worse than that of the CHL or MHL patients; they felt less satisfied with the effect of implantation and assessed the level of improvement as lower than the CHL or MHL patients. This confirms that any reduction in limitation need not correspond in any simple way with satisfaction. Our studies do not answer the key question—after BB implantation, which of the domains responsible for satisfaction causes differences in results between groups of patients with CHL, MHL, and SSD. In order to provide an answer, research should be continued with special attention to the issue of satisfaction. It is of interest, however, that the results of the satisfaction assessment correspond broadly to the clinical experiences of the authors.

Overall, based on an assessment under unaided and aided conditions of the limitations in activity and functioning caused by hearing loss, the BB system appears to have the technological capability to compensate for conductive hearing loss, mixed hearing loss (with a sensorineural component), as well as provide contralateral stimulation.

In all groups of patients, there were no correlations between the years of using the system and the degree of obtained benefits. It can therefore be assumed that the benefit after BB implantation is stable over time.

## 5. Conclusions

In this study, the PROMs indicated substantial benefit after BB implantation. In patients with CHL and MHL, no significant correlation was observed between bone conduction thresholds and air-bone gap (obtained by pure-tone audiometry before implantation) and the achieved benefits reported by the APHAB questionnaire after implantation. In all groups of users, there were also no correlations between the years of using the system and the degree of benefits obtained. In the APHAB questionnaire, no statistically significant differences were observed between the three groups of patients (CHL, MHL, and SSD). Differences were observed in the structured interview asking about the satisfaction with BB: satisfaction was larger in patients with CHL and MHL than with SSD, but this issue requires further investigation. It is concluded that the Bonebridge is an effective intervention in patients with CHL and MHL as well as with SSD.

## Figures and Tables

**Table 1:** Characteristics of the patients—number and frequency of occurrence (%).

		All Participants *n* = 81	CHL *n* = 25	MHL *n* = 36	SSD *n* = 20	Test Result
Sex	Women	39 (48.1)	12 (48.0)	17 (46.2)	10 (50.0)	χ^2^ = 0.04; *p* = 0.980
	Men	43 (51.9)	13 (52.0)	19 (52.8)	10 (50.0)	
Age	Range	18–74	18–67	18–74	19–72	*F* = 3.66; *p* = 0.030; *e^2^* = 0.08
	M [SD]	44.5 [16.2]	39.1 [16.1]	49.6 [15.9]	42.0 [14.7]	
Educational status	Primary	24 (29.6)	6 (24.0)	12 (33.3)	6 (30.0)	χ^2^ = 2.95; *p* = 0.567
	Secondary	26 (32.1)	9 (36.0)	13 (36.1)	4 (20.0)	
	University	31 (38.3)	10 (40.0)	11 (30.6)	10 (50.0)	
Marital status	Married, in relationship	50 (61.8)	16 (64.0)	21 (58.3)	13 (65.0)	χ^2^ = 0.32; *p* = 0.852
	Not in relationship	31 (38.2)	9 (36.0)	15 (41.7)	7 (35.0)	
Employment status	Employed	52 (64.2)	19 (76.0)	18 (50.0)	15 (75.0)	χ^2^ = 5.69; *p* = 0.058
	Unemployed	29 (35.8)	6 (24.0)	18 (50.0)	5 (25.0)	
Place of residence	Rural	22 (27.2)	5 (20.0)	12 (33.3)	5 (25.0)	χ^2^ = 1.39; *p* = 0.499
	Urban	59 (72.8)	20 (80.0)	24 (66.7)	15 (75.0)	
Use of a processor (hours/day)	Range	4–20	5–20	4–20	4–12	*F* = 5.18; *p* = 0.008; *e^2^* = 0.12
	M [SD]	9.8 [4.1]	9.7 [3.7]	11.0 [4.5]	7.6 [2.8]	
Use of BB (years)	Range	0.5–6.2	0.6–6.1	0.5–6.2	0.6–4.7	*F* = 9.87; *p* < 0.001; *e^2^* = 0.20
	M [SD]	2.8 [1.8]	3.1 [1.8]	3.7 [1.8]	2.0 [1.2]	

**Table 2:** Structured interview (questions and possible answers).

**1. Are you satisfied with the effect achieved?**
○ very satisfied ○ satisfied ○ somewhat between satisfied and dissatisfied ○ dissatisfied ○ very dissatisfied
**2. How do you rate your hearing (sound quality) with the Bonebridge system?**
○ very good ○ good ○ somewhat between good and bad ○ bad ○ very bad
**3. How do you rate the change in hearing when you use the processor compared to the state before the implant surgery?**
○ large improvement ○ minimal improvement ○ no change ○ minimal deterioration ○ large deterioration

**Table 3:** Unaided and aided scores on the APHAB questionnaire in the CHL and MHL group in terms of hearing impairment in the contralateral ear.

Hearing in Contralateral Ear		Unaided		Aided		*t*	*p*	
		*M*	*SD*	*M*	*SD*			*ES*
Normal hearing (*n* = 25)	EC	33.24	16.87	13.88	12.13	8.39	<0.001	1.68
	RV	42.16	18.15	25.16	16.28	6.93	<0.001	1.39
	BN	46.20	20.34	25.64	16.60	5.76	<0.001	1.15
	AV	23.64	21.16	31.88	23.27	2.28	0.032	0.46
	GS	40.54	16.99	21.56	13.85	8.26	<0.001	1.65
Mild/moderate HL (*n* = 25)	EC	58.32	19.98	21.80	12.96	8.43	<0.001	1.69
	RV	62.20	20.08	29.08	18.24	6.81	<0.001	1.36
	BN	61.68	16.87	26.32	16.55	8.92	<0.001	1.78
	AV	24.52	15.84	35.96	21.52	2.22	0.036	0.45
	GS	60.73	17.69	25.73	14.25	9.12	<0.001	1.82
Severe/very severe HL (*n* = 11)	EC	74.82	28.08	25.73	16.71	5.46	<0.001	1.65
	RV	79.73	25.75	39.36	19.04	4.59	0.001	1.38
	BN	76.73	28.11	35.55	18.50	4.87	0.001	1.47
	AV	21.82	25.57	41.45	22.90	2.01	0.073	0.61
	GS	77.09	24.53	33.55	16.44	5.68	<0.001	1.71

EC, Ease of Communication; RV, Reverberation; BN, Background Noise; AV, Aversiveness of Sounds; GS, Global Score (the average of the EC, RV, BN subscales); ES, effect size.

## Data Availability

The datasets used and/or analysed during the current study are available from the corresponding author on reasonable request.
